# Proteomic comparison of basal endosperm in maize *miniature*1 mutant and its wild-type *Mn*1

**DOI:** 10.3389/fpls.2013.00211

**Published:** 2013-06-25

**Authors:** Cecilia Silva-Sanchez, Sixue Chen, Ning Zhu, Qin-Bao Li, Prem S. Chourey

**Affiliations:** ^1^Proteomics, Interdisciplinary Center for Biotechnology Research, University of FloridaGainesville, FL, USA; ^2^Department of Biology, UF Genetics Institute, University of FloridaGainesville, FL, USA; ^3^USDA-Agricultural Research Service, Center for Medical, Agricultural and Veterinary EntomologyGainesville, FL, USA; ^4^Departments of Agronomy and Plant Pathology, University of FloridaGainesville, FL, USA

**Keywords:** proteomics, developing endosperm, iTRAQ, gene ontology, maize

## Abstract

Developing endosperm in maize seed is a major site for biosynthesis and storage of starch and proteins, and of immense economic importance for its role in food, feed and biofuel production. The basal part of endosperm performs a major role in solute, water and nutrition acquisition from mother plant to sustain these functions. The *miniature1* (*mn*1) mutation is a loss-of-function mutation of the *Mn*1-encoded cell wall invertase that is entirely expressed in the basal endosperm and is essential for many of the metabolic and signaling functions associated with metabolically released hexose sugars in developing endosperm. Here we report a comparative proteomic study between *Mn*1 and *mn*1 basal endosperm to better understand basis of pleiotropic effects on many diverse traits in the mutant. Specifically, we used iTRAQ based quantitative proteomics combined with Gene Ontology (GO) and bioinformatics to understand functional basis of the proteomic information. A total of 2518 proteins were identified from soluble and cell wall associated protein (CWAP) fractions; of these 131 proteins were observed to be differentially expressed in the two genotypes. The main functional groups of proteins that were significantly different were those involved in the carbohydrate metabolic and catabolic process, and cell homeostasis. The study constitutes the first proteomic analysis of basal endosperm cell layers in relation to endosperm growth and development in maize.

## Introduction

Proteomic analysis aims to measure the expression and modification of all proteins in an organism as a function of a number of variants, including environmental conditions, biotic or abiotic stresses, wild type and mutant genotypes, developmental stages etc. (Chen and Harmon, 2006; Lan et al., 2011; Neilson et al., 2011; Owiti et al., 2011). Advancement in mass spectrometry, genome sequencing and bioinformatics has been major impetuses to high-throughput analyses in proteome studies (Miernyk and Hajduch, 2011). Of many plant materials used for proteomic studies, developing seeds are of particular interest because of their economic significance; additionally, they undergo many distinctive phases where each stage is marked by unique cellular or metabolic activity. Among the most recent seed proteomic studies, Lee and Koh (2011) described rice grain profiles at three developmental stages against fully mature grains to show 52 categories out of a total of 4172 proteins. Koller et al. (2002) compared rice proteomes of leaf, root and seed showing 622 leaf-specific, 862 root-specific and 512 seed-specific proteins. The assignment of function based on BLAST searches showed that 360 proteins have no homology to known proteins; thus, these were labeled as rice-specific proteins. A similar study in maize endosperm (Méchin et al., 2007) at seven developmental stages showed a total of 632 proteins; of these 496 were assigned to functional identification. A relative increase in the levels of glycolytic enzymes as compared to TCA enzymes in this study confirmed the previously demonstrated anoxic conditions of the endosperm. It also pointed to a crucial role for pyruvate orthophosphate dikinase in maintaining starch-protein balance during endosperm development.

**Figure 1:**
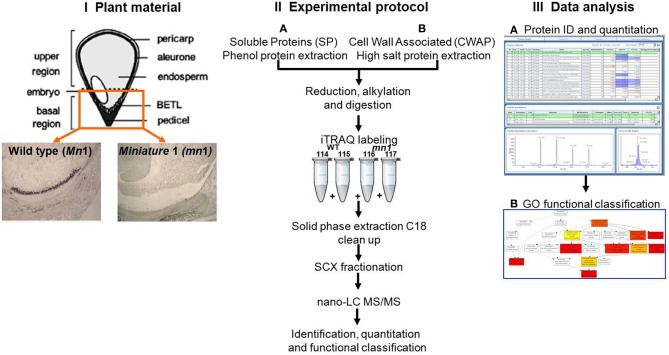
**Schematic experimental work flow: (I) Plant materials of *Mn*1 (wild type) and *mn*1 mutant enriched BETL regions; (II) SPs and CWAPs sample preparation, iTRAQ two-dimensional LC-MS/MS; and (III) data analysis**.

Although these data in maize are important, they do not take into account that an endosperm is a heterogeneous tissue comprised of at least four known cell types (Scanlon and Takacs, 2009). Of these four, one cell type, basal endosperm transfer layer (BETL), is unique to the basal 1/3rd part of the endosperm that is adjacent to the maternal pedicel (Figure 1), and is primarily engaged in acquisition of water, nutrients and solutes from the mother plant. In the context of basal endosperm, the *miniature*1 (*mn*1) seed mutant is of particular interest because it is a loss-of-function mutation of the *Mn*1 gene that encodes an endosperm-specific cell wall invertase (INCW2), which is entirely BETL-specific in its cellular localization (Cheng et al., 1996). Incoming sucrose to a sink tissue such as developing seed is irreversibly hydrolyzed by INCWs to glucose and fructose that is critical for numerous metabolic and signaling functions. In maize endosperm, the INCW2 is spatially and temporally the first enzyme to metabolize sucrose. Homozygous *mn*1 mutant is non-lethal due to a residual low level (<1% of the total activity) of INCW activity by the co-ortholog, *Incw*1 (Chourey et al., 2006). Not surprisingly, the *mn*1 mutant is associated with changes at both morphological (Miller and Chourey, 1992) and cellular (Vilhar et al., 2002) levels, which include retarded development of wall-in-growths (WIGs)—labyrinth-like growth in plasma membrane area believed to be essential to transport capacity of the BETL and the normal development of seed (Kang et al., 2009). The mutant is also associated with an altered IAA homeostasis (LeClere et al., 2008, 2010) and with greatly altered sugar metabolism, especially in the basal region that shows increased sucrose levels and greatly reduced hexose sugars due to its INCW2-deficiency (LeClere et al., 2010; Chourey et al., 2012). Reductions in both IAA and hexose sugars that are signaling molecules led to changes in the expression of several genes related to sucrose-starch and sucrose-energy metabolism in the *mn*1 mutant (Chourey et al., 2012). Xiong et al. (2011) reported 454 transcriptome sequencing of cDNAs to show 2473 unique sequences from *Mn*1 and *mn*1 BETLs. Functional annotation and categorization analyses of these cDNAs led them to conclude high abundance of transcripts related to mitochondrial activity, alkaloid biosynthesis, and various signaling processes of seed development. Transcriptomic studies however do not often provide a true representation of protein abundance due to the well-known phenomenon of poor concordance between RNA and the corresponding proteins as shown in seed filling in Arabidopsis (Hajduch et al., 2010).

The objectives of this study are 2-fold: (1) to analyze global proteome changes in the basal region of 12 DAP endosperm (i.e., BETL enriched) of the *mn*1 mutant relative to the *Mn*1 to describe or catalog proteins that may be metabolically co-regulated in response to the invertase deficiency. Reduced hexose levels may also activate many genes associated with untranslated protein response (UPR) stress. (2) In addition to soluble proteins (SPs), we also examined ionically-bound proteins presumably enriched for cell wall associate proteins (CWAPs). Because of the many cellular level changes in the *mn*1 basal endosperm (Kang et al., 2009) (most notably the changes in the WIG, which is predominantly comprised of initial primary cell wall and secondary cell wall formation during BETL maturation), we hypothesize that there will be interesting changes in the profiles of proteins associated with cell wall metabolism.

## Materials and methods

### Plant materials and chemicals

Immature maize (*Zea mays* L.) kernels of *Mn*1, and *mn*1 (Cheng and Chourey, 1999) in the W22 inbred line were harvested at 12 days after pollination (DAP). All plants were grown in the field and were self- or sib-pollinated. At the time of harvest kernels were individually excised from the ear with a paring knife, taking care to include undamaged base (pedicel) of each kernel. Excised kernels were flash frozen in liquid nitrogen and stored at −80°C until analysis. After removing embryos of each kernel, we used the basal 1/3 end of the endosperm because it is specifically enriched in the BETL cells, the sole site of the *Mn*1 expression and also a major zone for sucrose turn-over reactions, as discussed previously (LeClere et al., 2008). Chemicals were purchased from Fisher Scientific Inc., USA unless otherwise stated.

### Soluble protein extraction

A total of four biological samples, two each of the *Mn*1 and *mn*1 genotypes, and each sample comprised of 5 g of fresh tissue of basal 1/3 endosperm was dissected-out for SP extractions according to a previous method reported by Hurkman and Tanaka (1986) with minor modifications. Care was taken to save aliquots of the same kernel samples for the RNA analyses described below. Each frozen sample was ground into a fine powder with a pre-chilled mortar and pestle. A total of 3 mL of extraction buffer (0.1 M Tris-HCl pH 8.8, 10 mM EDTA, 0.2 M DTT, 0.9 M sucrose) and 3 mL of saturated phenol were added to homogenize the sample; and to extract proteins for 2 h at room temperature in constant agitation. The mixture was then centrifuged at 5000× g for 10 min at 4°C. The top clear phenol phase was removed and collected in a fresh tube. The remaining pellet was re-extracted with 3 mL buffered phenol solution and centrifuged again to recover the top clear phenol phase and combined with the previous one. The supernatant was precipitated with 5 volumes of ice cold 0.1 M ammonium acetate in 100% methanol overnight and centrifuged at 20000× g for 20 min at 4°C. The supernatant was discarded and the pellet was washed twice with 0.1 M ammonium acetate in 100% methanol, then washed twice with cold 80% acetone and finally with 70% of ethanol. The pellet was solubilized in a buffer containing 7 M urea, 2 M thiourea, 4% (w/v) CHAPS and 40 mM DTT. The samples were treated with benzonase (Novagen, Gibbstown, NJ) for 30 min and then ultracentrifuged at 34000 rpm for 30 min at 15°C. Supernatant was collected and 50 μ L aliquots were kept at −80°C until use.

### Cell wall associated protein extraction

In addition to the SP extraction, a high salt extraction was performed according to Cheng et al. (1996), to obtain cell wall associated proteins (CWAPs). Briefly, frozen kernels were homogenized in extraction buffer (50 mM Tris-maleate, pH 7.0 and 1 mM DTT) in a 1:10 (w/v) ratio by using a cooled mortar and pestle. The homogenate was centrifuged at 14000× g for 10 min. The supernatant was discarded and pellet was washed three times in the extraction buffer followed by a final resuspension in extraction buffer containing 1 M NaCl in a 1:2 (w/v) ratio. The salt suspension was vortexed for 30 min at 4°C and then centrifuged at 14000× g for 10 min. The supernatant was recovered and precipitated in a ratio 1:5 with 100% cold acetone overnight. Protein was recovered by centrifugation at 20000× g, 4°C for 30 min. The pellet was washed three times with cold acetone, followed by solubilization and storage as described for the SPs samples.

### iTRAQ labeling and LC-MS/MS

The SPs and CWAPs samples were quantified using an EZQ Protein Quantitation kit (Invitrogen; USA) (Zhu et al., 2010). A total of 100 μg of total protein from each sample was acetone precipitated. The samples were dissolved in 1% SDS, 100 mM triethylammonium bicarbonate, pH 8.5; then reduced, alkylated, trypsin (Promega, USA) digested and labeled according to manufacturer's instructions (ABsciex Inc. USA). The replicates of *Mn*1 samples were labeled with 114 and 115, whereas the replicates of *mn*1 samples were labeled with 116 and 117. Extra labels were quenched by adding 100 μ L of ultrapure water and left at room temperature for 30 min. After quenching, samples were mixed together and dried down in speedvac. The peptide mixtures were cleaned up with C18 spin columns according to manufacturer's instructions (ABsciex Inc. USA). The samples were then dissolved in strong cation exchange (SCX) solvent A (25% v/v ACN, 10 mM ammonium formate, pH 2.8) and injected to a Agilent HPLC 1100 system using a polysulfoethyl A column (2.1 × 100 mm, 5 μm, 300 Å, PolyLC, Columbia, USA). The peptides were eluted at a flow rate of 200 μ L/min with a linear gradient from 0 to 20% solvent B (25% v/v ACN, 500 mM ammonium formate) over 80 min, followed by a ramping up to 100% solvent B in 5 min and holding for 10 min. The peptides were detected at 214 nm absorbance and a total of 34 fractions were collected. Two independent iTRAQ experiments were conducted for the SPs and CWAPs samples.

Each SCX fraction was lyophilized in a speedvac and the resuspended in loading buffer (3% acetonitrile, 0.1% acetic acid, 0.01% TFA) and loaded onto a C18 capillary trap cartridge (LC Packings, USA) and then separated on a 15 cm nanoflow analytical C18 column (PepMap 75 μm id, 3 μm, 100 Å) at a flow rate of 200 nL/min on a Tempo nanoflow multidimensional LC system (ABsciex, USA). Solvent A composition was 3% ACN v/v, 0.1% acetic acid v/v; whereas solvent B was 97% ACN v/v, 0.1% acetic acid v/v. Peptides separation was performed with a linear gradient from 3 to 40% of solvent B for 2 h, followed by an increasing to 90% of solvent B in 10 min and hold for 10 min. Eluted peptides were introduced by ESI into a quadrupole time-of-flight mass spectrometer (QSTAR Elite MS/MS system, ABsciex Inc., USA). The source nebulizing gas and curtain gas were set at 12 and 20, respectively. Ion spray voltage was 2200V and the temperature was 80°C. The data were collected in the Information depended acquisition mode. A TOF-MS scan was done (m/z 300–1800, 0.25 s) automatically followed by the MS/MS scan (m/z 50–2000, 30–2000 ms) of the three abundant peptide ions per cycle. A 60 s exclusion was set for the former target ions. Automatic collision energy, automatic MS/MS accumulation and dynamic exclusion were selected in Analyst QS software.

### Protein identification, quantification, and functional classification

The identification and quantification of proteins were performed using ProteinPilot™ Software 4.0. The database was UniProt for maize (March 29, 2011; 84916 entries). The searching parameters were set as iTRAQ peptide label, cysteine alkylation with methyl methanethiosulfonate, trypsin digestion, identification focus for biological modifications and BIAS correction. The unused score threshold was set to >1.3 (equivalent to 95% confidence or better) and *p*-value < 0.05 to ensure that quantitation was based on at least three unique peptides. The proteins were considered for validation if they were significant in both independent experiments.

For functional classification, the protein lists were analyzed according to the Gen Ontology (GO) using the Singular Enrichment Analysis (SEA) tool (http://bioinfo.cau.edu.cn/agriGO/) (Du et al., 2010) for biological, molecular and cellular function classification, considering a background database list of *Zea mays* and the Plant Slim algorism available in the agriGO. The results were exported directly to REVIGO (Supek et al., 2011) in order to visualize the clusters of the enriched GO terms. The KEGG encyclopedia was used for the identification of pathways (http://www.genome.jp/kegg) (Kanehisa et al., 2012).

### RNA extraction, cDNA synthesis, and quantitative real-time PCR (qRT-PCR)

All RNA studies were done on the same sample papers as those used in the above iTRAQ analyses. RNA extraction, cDNA synthesis, and qRT-PCR were done as described previously (Chourey et al., 2010; LeClere et al., 2010). Briefly, total RNA was extracted from each sample of 100 mg tissue (a total of four samples, two for each genotype) and treated with DNA-free DNase I (Ambion, TX, USA). Purified RNA samples were quantified using a Nanodrop ND-4000 Spectrophotometer (Thermo Scientific, DE, USA) and evaluated using an Agilent 2100 Bioanalyzer (Agilent Technologies, CA, USA). First-strand cDNA was synthesized with 5 μg of purified RNA using reverse transcriptase (RT) Superscript III (Invitrogen, CA, USA). qRT-PCR assays for each target were performed via MyiQ ver. 2.0 with iQ ver. 5.0 (Bio-Rad, CA, USA), using the primers (Table S1) and SYBER green dye method as previously described (Koh et al., 2010). Our previous studies have shown that absolute q-PCR method, as used here, is a reliable estimate of gene expression based on several genes and an excellent concordance between the low resolution Northern blot hybridization and absolute q-PCR values (Chourey et al., 2010; LeClere et al., 2010).

## Results and discussion

### Identification of soluble proteins (SPs) and cell wall associated proteins (CWAPs) in BETL enriched regions

**Figure 2:**
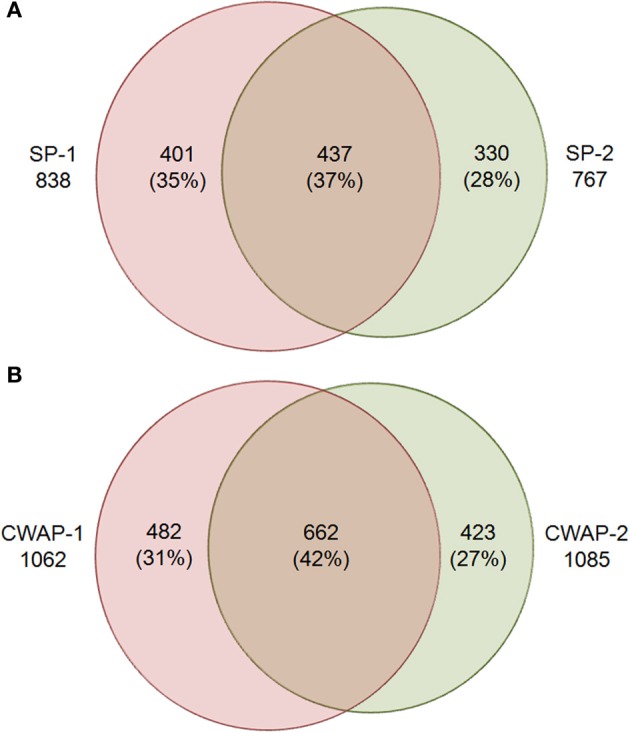
**Venn diagram showing the overlap of protein identities in two biological replicates for (A) soluble proteins (SPs), SPs-1 and SPs-2 are biological replicates; and (B) cell wall associated proteins (CWAPs) in two biological replicates**.

One of the main functions of the basal part of maize endosperm is to acquire and transfer of nutrients and water from maternal plant to developing seed through the BETL localized in the basal region. The lack of *Mn*1-encoded INCW2 protein in the *mn*1 BETL has led to many pleiotropic changes in the mutant relative to the *Mn*1 (Chourey et al., 2012). The 12 DAP stage used in this study is ideal as it marks the highest levels of INCW2 activity (Cheng et al., 1996), and a major point of developmental and metabolic switch from cell division, cell elongation phase to the initiation of storage phase which is expected to be associated with proteomic changes in the *Mn*1 and the mutant *mn*1 endosperm. Here we have used a multiplex iTRAQ approach in the systematic identification and quantification of the expression levels of both soluble and CWAPs in the two genotypes, *Mn*1 and *mn*1. The experimental strategy based on the use of LC-MS/MS and the GO functional classification is depicted in the Figure 1. We identified a total of 1168 SPs in two independent experiments with a 1.3 unused score threshold (95% confidence) (Table S2). A total of 437 proteins were identified in both experiments representing a 38% overlap, and there were 401 (34%) and 330 (28%) unique proteins for experiments 1 and 2, respectively (Figure 2A). For CWAPs, there were 1567 identifications in two independent experiments (Table S2). A total of 662 proteins were identified in both experiments, representing a 42% overlap, and there were 482 (31%) and 423 (27%) unique proteins identified in experiments 1 and 2, respectively (Figure 2B). When comparing SPs and CWAPs protein identifications, only 36% of the IDs were shared between the two sample types, suggesting the complementary nature of the different extractions (Tables S2, S3). Reproducibility of protein identification is known to be affected in replicates of iTRAQ experiments, and any given run may identify only a subset of relevant peptides in a complex mixture causing variation among analytical runs (Lee and Koh, 2011; Owiti et al., 2011). The overlap showed for SPs, CWAPs, and in between the two independent replicate experiments was comparable to previous reports (Chen et al., 2007) that show the range of 25–40% in similar iTRAQ analyses.

### Functional annotation of identified proteins

**Figure 3:**
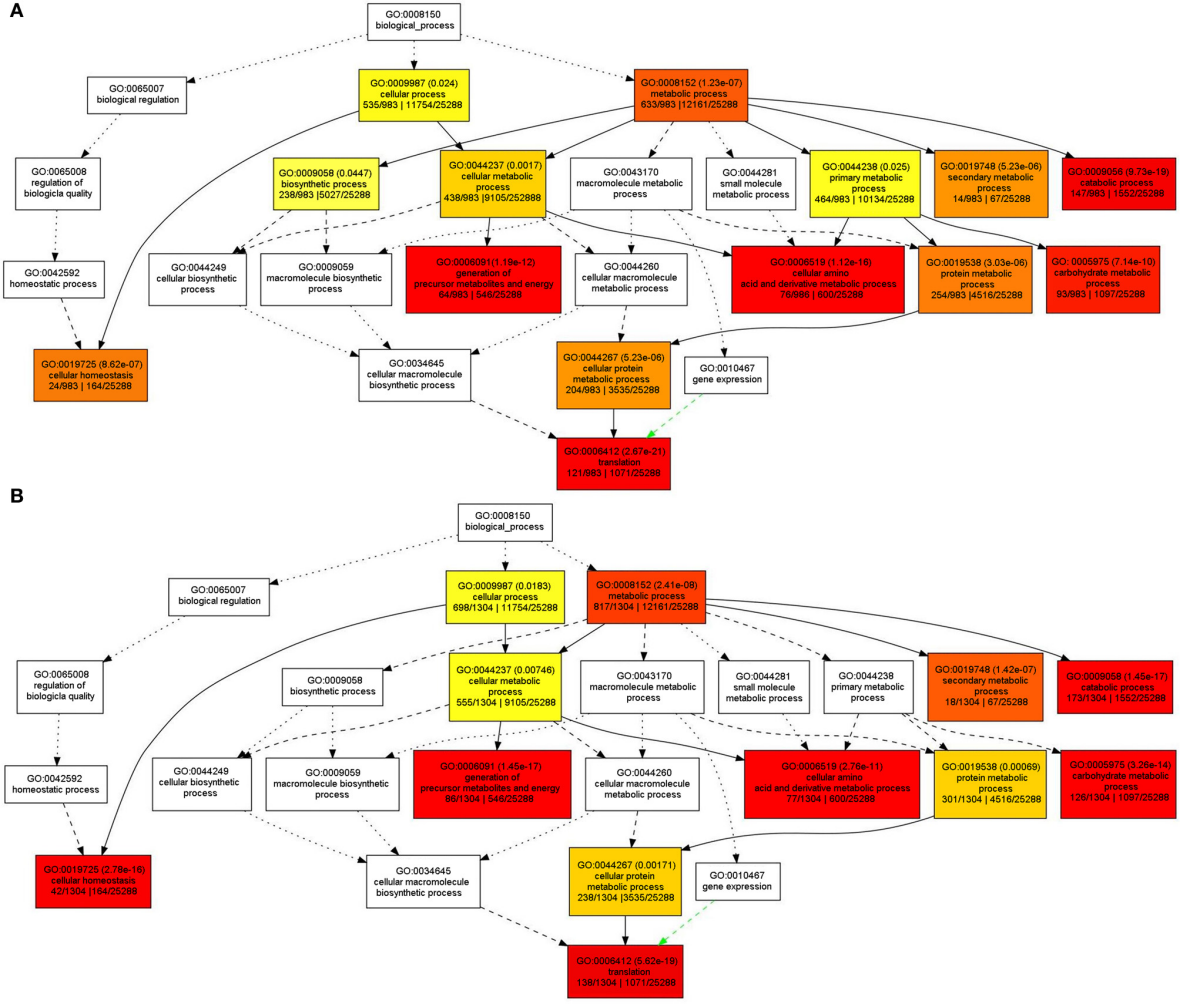
**GO classification of enriched biological functions in (A) SPs and (B) CWAPs with agriGO tool kit**.

In order to understand the functions of the identified SPs and CWAPs and their related processes, a Gene Ontology (GO) classification was performed using the agriGO Single Enrichment Analysis (SEA) tool (http://bioinfo.cau.edu.cn/agriGO). A plant GO slim type was used for the SPs in which 984 (84%) of the 1168 proteins were annotated. The results showed 14 GO terms enriched for biological process, 5 GO terms for molecular function, and 17 GO terms for cellular component (Table S4). In general, the GO terms that were significantly enriched are related to cellular and metabolic processes. Specifically those belonging to primary and secondary metabolic process, catabolic process, carbohydrates metabolic process and cellular homeostasis (Figure 3A) that could be attributed to the INCW2-deficiency in the basal region of *mn*1 seeds. Following the same strategy as in the analysis of SPs, a total of 1034 (83%) entries of the 1567 CWAPs were annotated; i.e., 12 GO terms enriched for biological process, 5 GO terms for molecular function, and 18 GO terms for cellular component (Table S5). The terms for biosynthetic process and primary metabolic process were not found in the CWAPs (Figure 3B). Similar proteomic studies in developing grains of rice also show the predominant role of carbohydrate metabolic processes in seed development (Lee and Koh, 2011).

GO classification results are usually displayed as a tree form, in which if one of the end point of the branch is enriched, the previous nodes to the branch will be enriched as well, leading to some redundancy. The REVIGO tool was used to analyze the GO enriched terms in order to reduce the redundancy and allow visualization of the most informative common ancestor nodes. Figure S1 shows the enriched biological functions in the SPs and CWAPs. Interestingly, both fractions are strikingly similar. With the exception of biological process and primary metabolic process in SPs, both analyses revealed the translation process as the main one affected by the mutation and directly related to the cellular homeostasis process (Figure S1A,B). These results suggest that multiple biological processes were affected in the *mn*1 mutant and the analysis of both fractions suggests a complementary nature of the two protein fractions in the basal endosperms.

### Differentially expressed proteins in mn1 maize basal endosperm

Differential expression of proteins in any two independent experiments by iTRAQ is considered statistically significant if (i) they show an average change of 20% or greater, (ii) a *p-value* less than 0.05, and (iii) obtained from measuring at least three unique peptides to ensure significant quantitative changes (Ali et al., 2010; Zhu et al., 2010). With these criteria, we retrieved a total of 100 SPs that were differentially expressed; among which 15 showed decreased and 85 showed increased levels in the mutant as compared to the *Mn*1 (Table 1). Among the CWAPs, a total of 91 proteins were differentially expressed, of which 33 showed decreased and 58 showed increased levels in the mutant as compared to the *Mn*1 (Table 2). Of the total 191 differentially expressed proteins in both SPs and CWAPs samples, only 15 proteins were common to both fractions (Table S6), suggesting that extraction protocol was rigorous in allowing most of the proteins to be fractionated. It should be noted that the salt extraction protocol was effective in releasing ionically bound CWAPs. All shared proteins exhibited similar expression ratios except for B4G0K5, which was decreased in the SPs and increase in the CWAPs. This protein has no known functions and no annotations could be found to assign or hypothesize its function. It is not surprising that certain CWAPs were also released into the soluble fraction during the homogenization in low salt concentration of buffer, as shown previously for the INCW2 (Carlson and Chourey, 1999).

**Table 1:** **Proteins identified as differentially expressed from iTRAQ LC-MS/MS of soluble proteins (SPs)**.

**Accession**	**Name**	**Experiment 1**		**Experiment 2**	
		***mn*1/*Mn*1**	***SD***	***mn*1/*Mn*1**	***SD***
B6SZ52	Putative uncharacterized protein	**0.35**	**0.04**	**0.42**	**0.02**
Q9SPJ9, Q9SPK0	^a^Cell wall invertase 2	**0.37**	**0.05**	**0.30**	**0.02**
B6EBQ2	Indole-3-acetic acid amido synthetase	**0.52**	**0.01**	**0.77**	**0.01**
B4G0K5	Putative uncharacterized protein	**0.58**	**0.01**	0.81	0.01
B6TBZ8	Alanine aminotransferase 2	**0.64**	**0.01**	0.85	0.02
B6TEC1	Sorbitol dehydrogenase	**0.67**	**0.00**	0.80	0.05
Q43706	Sus1 protein	**0.71**	**0.01**	0.93	0.03
Q548K3	Farnesyl diphosphate synthase	**0.74**	**0.02**	1.01	0.02
Q5EUE1	^b^Protein disulfide isomerase	**0.76**	**0.02**	0.96	0.02
B7ZXK1	Aspartate aminotransferase	**0.76**	**0.00**	0.92	0.01
E9NQE5	Pyruvate orthophosphate dikinase 1	**0.78**	**0.02**	0.90	0.03
B6T4Q5	Pro-resilin	0.91	0.00	**1.32**	**0.09**
Q6VWF6	O-methyltransferase (Fragment)	0.92	0.04	**1.20**	**0.02**
C0HHC4	Nucleoside diphosphate kinase	0.92	0.03	**1.26**	**0.05**
C4J473	Similar to oligopeptidase A-like from *Oryza sativa* (Q6K9T1)	0.93	0.01	**1.25**	**0.01**
B6TRW8	Dihydrolipoyllysine-residue succinyltransferase component of 2-oxoglutarate dehydrogenase complex	0.95	0.02	**1.32**	**0.00**
Q84TL6	^c^Legumin-like protein	0.96	0.01	**1.30**	**0.01**
F8UV61	Cell division cycle protein 48 (Fragment)	0.96	0.04	**1.23**	**0.03**
B6TNF1	Calnexin	0.98	0.08	**1.24**	**0.01**
C0P2V1	Similar to leucine aminopeptidase 2, chloroplastic from *Oryza sativa* (Q6K669)	0.98	0.05	**1.35**	**0.03**
C0HGV5	Enolase	1.01	0.02	**1.46**	**0.08**
Q5EUD6	^b^Protein disulfide isomerase	1.02	0.01	**1.32**	**0.04**
B7ZWY9	Citrate synthase	1.02	0.02	**1.35**	**0.03**
B4G0S0	Similar to UMP synthase from *Zea mays* (Q9LKI4)	1.03	0.07	**1.28**	**0.04**
Q6R987	ATP synthase subunit alpha	1.03	0.01	**1.23**	**0.04**
C0PL01	Similar to T-complex protein 1 subunit gamma from *Zea mays* (B6UCD0)	1.03	0.01	**1.30**	**0.01**
B6SXV4	Peroxisomal fatty acid beta-oxidation multifunctional protein	1.03	0.02	**1.28**	**0.04**
B6U0V6	Endoplasmin	1.03	0.02	**1.33**	**0.04**
B4FNM4	Similar to 60S acidic ribosomal protein P0 from *Zea mays* (O24573)	1.03	0.02	**1.42**	**0.00**
B4FH47	Similar to USP family protein from *Zea mays* (B6TC12)	1.04	0.05	**1.46**	**0.02**
Q8S4W9	Pyruvate decarboxylase	1.04	0.05	**1.23**	**0.04**
B4FFH8	Adenosine kinase 2	1.04	0.00	**1.31**	**0.02**
B6T856	L-lactate dehydrogenase	1.05	0.02	**1.35**	**0.02**
C4J5G3	Similar to dihydrolipoyl dehydrogenase from *Sorghum bicolor* (C5XIY9)	1.05	0.01	**1.34**	**0.00**
B4FGL3	Similar to mitochondrial ATP synthase from *Zea mays* (B6TCR9)	1.05	0.04	**1.39**	**0.01**
B6TDE0	NADP-dependent oxidoreductase P1	1.07	0.03	**1.42**	**0.04**
B6TJB6	Proteasome subunit alpha type	1.07	0.00	**1.39**	**0.06**
C4J4E4	Similar to cytosolic monodehydroascorbate reductase from *Oryza sativa* (Q9XFZ3)	1.08	0.04	**1.37**	**0.04**
B6TI78	Peptidyl-prolyl isomerase	1.08	0.05	**1.44**	**0.00**
Q5GAU1	Putative alanine aminotransferase	1.09	0.01	**1.35**	**0.00**
B6UHU1	Catalase	1.09	0.03	**1.26**	**0.00**
B6U4A3	Heat shock 70 kDa protein	1.10	0.02	**1.27**	**0.09**
B8A1R8	Similar to 5-methyltetrahydropteroyltriglutamate-homocysteine methyltransferase from *Zea mays* (B6UF55)	1.11	0.03	**1.27**	**0.03**
B4G080	Caffeoyl-CoA O-methyltransferase 1	1.11	0.01	**1.87**	**0.07**
B4FCK0	USP family protein	1.11	0.01	**1.37**	**0.02**
B6T3G4	40S ribosomal protein S19	1.12	0.02	**1.45**	**0.04**
B8A2W6	Putative uncharacterized protein	1.12	0.04	**1.55**	**0.01**
Q9LLB8	Exoglucanase	1.13	0.06	**1.41**	**0.03**
Q5EUD7	^b^Protein disulfide isomerase	1.14	0.03	**1.49**	**0.04**
C0PK05	Similar to lactoylglutathione lyase from *Zea mays* (B6TPH0)	1.17	0.02	**1.54**	**0.01**
C0P820	Similar to 3-ketoacyl-CoA thiolase 2, peroxisomal, putative, expressed from *Oryza sativa* (Q94LR9)	1.18	0.04	**1.38**	**0.03**
B6SXW8	RuBisCO large subunit-binding protein subunit alpha	**1.21**	**0.01**	**1.60**	**0.00**
B8A1M2	Similar to thioredoxin-like proteon 5 from *Zea mays* (B6TP19)	**1.22**	**0.01**	**1.79**	**0.06**
C0HF77	Putative uncharacterized protein	**1.22**	**0.05**	**1.35**	**0.01**
B6UAU9	Rhicadhesin receptor	**1.23**	**0.04**	**1.47**	**0.02**
B6UAK0	6-phosphogluconolactonase	**1.28**	**0.06**	**1.71**	**0.00**
Q8W2B6	UDP-glucosyltransferase BX9	**1.28**	**0.04**	**1.54**	**0.02**
Q84TL7	^c^Legumin-like protein	**1.28**	**0.01**	**1.50**	**0.03**
B6T522	40S ribosomal protein S14	**1.30**	**0.02**	**1.46**	**0.04**
B8A326	Similar to plasma membrane ATPase from *Oryza sativa* (Q7XPY2)	**1.33**	**0.03**	**1.60**	**0.04**
B6TVW2	Germin-like protein subfamily 1 member 17	**1.37**	**0.09**	**1.62**	**0.00**
B6T4C7	Peptidyl-prolyl cis-trans isomerase	**1.42**	**0.09**	**1.88**	**0.03**
C4J9Y2	Similar to stem-specific protein TSJT1 from *Zea mays* (B4FQW0)	**1.42**	**0.02**	**2.08**	**0.06**
C4J409	Putative uncharacterized protein	**1.44**	**0.01**	**1.87**	**0.09**
B8A3M0	^d^Glutamine synthetase	**1.45**	**0.07**	**1.86**	**0.06**
B4G218	Putative uncharacterized protein	**1.51**	**0.02**	**2.24**	**0.03**
B4FTP4	Putative uncharacterized protein	**1.59**	**0.06**	**2.12**	**0.05**
B4FT23	14-3-3-like protein	**1.63**	**0.05**	**2.09**	**0.10**
B4FG65	Similar to catalytic/hydrolase from *Zea mays* (B6U0A3)	**2.43**	**0.09**	**2.83**	**0.15**
B4FFQ0	Thioredoxin	**2.68**	**0.13**	**2.93**	**0.09**
B6SHX0	5a2 protein	**0.46**	**0.00**	ND	ND
Q43359	Cytosolic glyceroldehyde-3-phosphate dehydrogenase GAPC4	**0.62**	**0.04**	ND	ND
B4FK84	Similar to glutathione transferase III(A) from *Zea mays* (Q9ZP62)	**0.79**	**0.00**	ND	ND
B6TM55	^e^APx1-cytosolic ascorbate peroxidase	**1.23**	**0.04**	ND	ND
B4FFJ4	Similar to alpha-galactosidase from *Oryza sativa* (Q9FXT4)	**1.42**	**0.09**	ND	ND
B6TYM9	^f^Vignain	**1.61**	**0.04**	ND	ND
C5JA67	BETL-9 protein	ND	ND	**0.45**	**0.01**
B6TP93	Fructokinase-2	ND	ND	**1.20**	**0.02**
B4FBF4	Serine hydroxymethyltransferase	ND	ND	**1.24**	**0.01**
B6TJM5	26S protease regulatory subunit 6A	ND	ND	**1.26**	**0.02**
B4FZV6	Similar to 26S protease regulatory subunit 7 from *Oryza sativa* (Q9FXT9)	ND	ND	**1.26**	**0.00**
B6SJ21	Guanine nucleotide-binding protein beta subunit-like protein	ND	ND	**1.27**	**0.02**
B6TMX0	Pyruvate kinase	ND	ND	**1.32**	**0.07**
Q94G64	T-cytoplasm male sterility restorer factor 2	ND	ND	**1.34**	**0.02**
C0PHP3	Similar to 60 kDa chaperonin beta subunit from *Oryza sativa* (Q6ZFJ9)	ND	ND	**1.35**	**0.03**
B6T782	40S ribosomal protein SA	ND	ND	**1.36**	**0.03**
C0PGM6	Similar to 26S protease regulatory subunit S10B from *Zea mays* (B4FTV9)	ND	ND	**1.37**	**0.04**
B4FBY6	Similar to caffeoyl CoA 3-O-methyltransferase from *Zea mays* (Q7X6T0)	ND	ND	**1.40**	**0.01**
B4G1D2	CBS domain protein	ND	ND	**1.49**	**0.07**
B6SIF5	Translationally-controlled tumor protein	ND	ND	**1.49**	**0.03**
C0P397	Similar to adenine phosphoribosyltransferase 2 from *Zea mays* (B6TGM2)	ND	ND	**1.49**	**0.03**
B8A0Q6	Similar to succinate dehydrogenase flavoprotein subunit, mitochondrial from *Zea mays* (B6U124)	ND	ND	**1.51**	**0.04**
B7ZZ39	Glutamate dehydrogenase	ND	ND	**1.52**	**0.04**
B6UB73	^e^APx1-cytosolic ascorbate peroxidase	ND	ND	**1.58**	**0.03**
B4G1X1	Putative uncharacterized protein	ND	ND	**1.59**	**0.05**
B6SS31	Putative uncharacterized protein	ND	ND	**1.60**	**0.03**
B9TSW1	^d^Glutamine synthetase	ND	ND	**1.61**	**0.04**
B7ZXW9	Similar to alpha-galactosidase from *Oryza sativa* (Q9FXT4)	ND	ND	**1.62**	**0.01**
E7DDV3	Peroxisomal-CoA synthetase	ND	ND	**1.95**	**0.06**
B6TLR1	^f^Vignain	ND	ND	**2.25**	**0.06**

Values shown in bold indicate protein fold changes lower than 0.8 or higher than 1.2 and p-value < 0.05.

Ratios mn1/Mn1 are average values of the two independent replicates, each quantified with at least three unique peptides.

a Isoform with a single amino acid substitution, quantification was done only with the common peptides.

b,c,d,e,f Isoforms detected in the experiment.

**Table 2:** **Proteins identified as differentially expressed from iTRAQ LC-MS/MS of cell wall associated proteins (CWAPs)**.

**Accession**	**Name**	**Experiment 1**		**Experiment 2**	
		***mn*1/*Mn*1**	***SD***	***mn*1/*Mn*1**	***SD***
Q9SPK0	Cell wall invertase 2	**0.38**	**0.02**	**0.27**	**0.00**
Q19VG6	Major latex protein 22	**0.45**	**0.00**	**0.51**	**0.02**
Q4FZ46	Cystatin	**0.48**	**0.00**	**0.54**	**0.01**
B6T6D4	Peroxidase 27	**0.52**	**0.02**	**0.53**	**0.00**
C0P6F8	Similar to sucrose synthase 2 from *Zea mays* (P49036)	**0.57**	**0.00**	**0.71**	**0.01**
Q9FER8	HMGI/Y protein	**0.57**	**0.01**	**0.44**	**0.01**
B4FSS8	Putative uncharacterized protein	**0.57**	**0.03**	**0.62**	**0.00**
B4G004	Similar to beta-glucosidase 7 from *Oryza sativa* (Q75I93)	**0.63**	**0.04**	**0.60**	**0.04**
C4JC17	Putative uncharacterized protein	**0.63**	**0.09**	**0.66**	**0.00**
B6TDW7	Secretory protein	**0.66**	**0.03**	**0.59**	**0.02**
B6T4G7	Fibrillarin-2	**0.68**	**0.02**	0.82	0.00
B6TPA2	Putative uncharacterized protein	**0.68**	**0.01**	**0.57**	**0.00**
B4FHA8	Similar to histone deacetylase HDT2 from *Zea mays* (Q9M4U5)	**0.69**	**0.03**	**0.76**	**0.12**
Q4FZ49	Putative cystatin	**0.69**	**0.02**	**0.75**	**0.00**
B7ZXK1	Aspartate aminotransferase	**0.73**	**0.01**	**0.75**	**0.02**
C0P8K0	Similar to 3-hydroxybutyryl-CoA dehydratase from *Zea mays* (B6TF44)	**0.75**	**0.00**	**0.77**	**0.05**
B6TKT8	Esterase	**0.76**	**0.02**	**0.72**	**0.03**
B6TS72	U2 small nuclear ribonucleoprotein A	**0.76**	**0.03**	**0.79**	**0.02**
B4FT31	Similar to chloride intracellular channel 6 from *Zea mays* (B6UCX2)	**0.76**	**0.02**	**0.72**	**0.02**
E9NQE5	Pyruvate orthophosphate dikinase 1	**0.77**	**0.02**	**0.74**	**0.00**
B6TIQ8	ATP/GTP binding protein	**0.78**	**0.00**	0.85	0.05
B6TE01	Myosin-like protein	0.80	0.07	**0.76**	**0.01**
B6THU9	Peroxidase 39	0.81	0.01	**0.76**	**0.00**
B6SLX1	Chaperonin	0.85	0.04	**0.79**	**0.02**
B6UDP8	Protein CYPRO4	0.97	0.01	**0.76**	**0.01**
B6TG70	Mitochondrial-processing peptidase beta subunit	1.01	0.01	**1.28**	**0.02**
Q9SAZ6	Phosphoenolpyruvate carboxylase	1.10	0.06	**1.20**	**0.02**
B4FER8	Acyl-CoA-binding protein	1.13	0.03	**1.23**	**0.05**
B4FNM4	Similar to 60S acidic ribosomal protein P0 from *Zea mays* (O24573)	1.18	0.03	**1.35**	**0.01**
B6TRV8	Eukaryotic initiation factor 5C CG2922-PF, isoform F	**1.20**	**0.03**	1.19	0.06
C0P8C6	Similar to TCP-1/cpn60 chaperonin from *Oryza sativa* (C6F1N7)	**1.20**	**0.12**	1.10	0.02
Q43712	Calcium-binding protein	**1.20**	**0.01**	**1.32**	**0.03**
B8A0J2	Putative uncharacterized protein	**1.21**	**0.02**	1.13	0.02
B6SPX4	Tubulin alpha-3 chain	**1.22**	**0.02**	1.15	0.03
C0P2V1	Similar to leucine aminopeptidase 2, chloroplastic from *Oryza sativa* (Q6K669)	**1.23**	**0.02**	1.03	0.06
B4FTP4	Putative uncharacterized protein	**1.26**	**0.01**	**1.65**	**0.05**
C0PDB6	Similar to 3-N-debenzoyl-2-deoxytaxol N-benzoyltransferase from *Zea mays* (B6TJ78)	**1.27**	**0.00**	**1.39**	**0.08**
Q9AXG8	Lipoxygenase	**1.28**	**0.01**	1.11	0.00
Q9M588	Prohibitin	**1.30**	**0.02**	**1.21**	**0.08**
B6TB97	40S ribosomal protein S3	**1.31**	**0.00**	**1.25**	**0.02**
B2ZAF9	Malate dehydrogenase	**1.31**	**0.04**	**1.22**	**0.02**
C0P4D8	Similar to dynamin-2A from *Zea mays* (B6UEQ3)	**1.32**	**0.03**	**1.30**	**0.04**
C0PH85	Similar to tubulin beta-3 chain from *Eleusine indica* (Q9ZPN8)	**1.32**	**0.01**	**1.33**	**0.04**
C0P406	Putative uncharacterized protein	**1.33**	**0.07**	1.12	0.04
Q6R9G1	NADH dehydrogenase subunit 7	**1.33**	**0.02**	1.13	0.03
Q8S4W9	Pyruvate decarboxylase	**1.33**	**0.02**	1.13	0.00
B6TYX3	USP family protein	**1.35**	**0.04**	**1.25**	**0.02**
C0PHK6	Similar to dynamin-related protein 1C from *Zea mays* (B6U1C4)	**1.38**	**0.09**	**1.80**	**0.22**
C0HFU7	Phospholipase D	**1.38**	**0.05**	**1.35**	**0.08**
Q5EUD5	Protein disulfide isomerase	**1.39**	**0.04**	**1.23**	**0.01**
B4FV87	Jasmonate-induced protein	**1.45**	**0.03**	**1.39**	**0.03**
B4FUE0	GTP-binding protein PTD004	**1.45**	**0.02**	**1.40**	**0.02**
B4FCQ4	Cytochrome c oxidase subunit	**1.46**	**0.02**	**1.32**	**0.02**
C0PHF3	Similar to alpha-glucosidase like protein from *Hordeum vulgare* (B5U8Z1)	**1.47**	**0.05**	1.17	0.03
B4G0K5	Putative uncharacterized protein	**1.48**	**0.03**	1.19	0.05
B6U0V6	Endoplasmin	**1.52**	**0.00**	**1.36**	**0.02**
B6TC70	Acid phosphatase	**1.52**	**0.03**	**1.86**	**0.11**
B4F9U9	Putative uncharacterized protein	**1.59**	**0.01**	**1.44**	**0.00**
B6U4J8	Dynamin-related protein 1A	**1.60**	**0.00**	1.19	0.04
B4FX40	Similar to cysteine proteinase 1 from *Zea mays* (Q10716)	**1.62**	**0.01**	**1.56**	**0.02**
Q9M585	Stomatin-like protein	**1.64**	**0.01**	1.19	0.04
C4J409	Putative uncharacterized protein	**1.67**	**0.04**	**1.90**	**0.21**
C0HF77	Putative uncharacterized protein	**1.69**	**0.04**	**1.87**	**0.09**
B6T887	Salt tolerance protein	**1.76**	**0.09**	**1.81**	**0.01**
B4FKH7	Cytochrome b5	**2.03**	**0.06**	**1.75**	**0.05**
B4FSK9	Peroxidase 1	**2.23**	**0.13**	**1.48**	**0.04**
B6SHX0	5a2 protein	**0.53**	**0.02**	ND	ND
Q948J8	Uncleaved legumin-1	**0.63**	**0.02**	ND	ND
B4FSU9	Hydrolase, hydrolyzing O-glycosyl compounds	**0.68**	**0.01**	ND	ND
B4FP25	^a^40S ribosomal protein S19	**0.70**	**0.01**	ND	ND
C0PDM0	Similar to vaculor H+-pyrophosphatase from *Sorghum bicolor* (D9IG65)	**0.72**	**0.03**	ND	ND
C0P5P9	Glycylpeptide N-tetradecanoyltransferase	**0.78**	**0.02**	ND	ND
O49010	Herbicide safener binding protein	**0.78**	**0.04**	ND	ND
B6UCD0	T-complex protein 1 subunit gamma	**1.26**	**0.04**	ND	ND
B8A2Z3	Coatomer subunit gamma	**1.28**	**0.05**	ND	ND
C0P531	Similar to 26S proteasome non-ATPase regulatory subunit 3 from *Zea mays* (B6TBG8)	**1.34**	**0.05**	ND	ND
C0P5E7	Putative uncharacterized protein	**1.34**	**0.04**	ND	ND
B4G1Q6	Similar to protein Z from *Zea mays* (B6TS23)	**1.37**	**0.02**	ND	ND
B4FQK5	Eukaryotic peptide chain release factor subunit 1-1	**1.47**	**0.01**	ND	ND
C0PJ26	Similar to DEAD-box ATP-dependent RNA helicase 15 from *Oryza sativa* (Q5JK84)	**1.63**	**0.05**	ND	ND
B4FUK7	Putative uncharacterized protein	**2.04**	**0.08**	ND	ND
B4FG65	Similar to catalytic/hydrolase from *Zea mays* (B6U0A3)	**2.42**	**0.04**	ND	ND
C5JA67	BETL-9 protein	ND	ND	**0.49**	**0.00**
Q946V2	Legumin 1	ND	ND	**0.60**	**0.01**
B6T3G4	^a^40S ribosomal protein S19	ND	ND	**0.69**	**0.01**
B4FGC8	40S ribosomal protein S12	ND	ND	**0.75**	**0.00**
B4F7T9	Putative uncharacterized protein	ND	ND	**0.75**	**0.01**
C0PP73	Similar to ADP-ribosylation factor from *Triticum aestivum* (Q76ME3)	ND	ND	**1.21**	**0.00**
B4G178	ADP, ATP carrier protein	ND	ND	**1.27**	**0.09**
B4FRG8	Similar to mitochondrial 2-oxoglutarate/malate carrier protein from *Zea mays* (B6T8M6)	ND	ND	**1.30**	**0.01**
O24449	Translational initiation factor eIF-4A	ND	ND	**1.56**	**0.02**

Values shown in bold indicate protein fold changes lower than 0.8 or higher than 1.2 and p-value < 0.05.

Ratios mn1/Mn1 are average values of the two independent replicates, each quantified with at least three unique peptides.

a Isoforms found in the experiment.

Table 3 shows GO annotations for SPs that were differentially expressed in the two genotypes. It includes proteins in catabolic process, cellular homeostasis and carbohydrate metabolism as biological functions through SEA described in the Materials and Methods. The differentially expressed CWAPs did not show any significant enrichment for biological function (Table 3). In general, the main processes that are affected due to the lack of INCW2 are related to the compensation of the energy supply via the carbohydrate metabolic process and establishment of normal activity in the cell through the catabolic process. Similar proteomic study in developing rice grain shows that carbohydrate metabolic process, transport, and localization are the main cellular events (Lee and Koh, 2011).

**Table 3:** **Differentially expressed SPs with their predicted biological functions**.

**Biological process**	**Accession**	**Name**	***mn*1/*Mn*1**	***SD***	**KEGG identifier**	**Pathway identifier**
Catabolic process (GO:0009056)	C4J473	Putative uncharacterized protein	1.09	0.23	NF	NF
	C0P2V1	pco101682(581); LOC100381923; K01255 leucyl aminopeptidase [EC:3.4.11.1]	1.17	0.26	zma:100381923	zma00480, zma01100
	B6UHU1	Catalase isozyme B; K03781 catalase [EC:1.11.1.6]	1.17	0.12	zma:100857004	zma00380, zma00630, zma01100, zma01110, zma04146
	B7ZWY9	Putative uncharacterized protein (Pfam: citrate synthase domain)	1.18	0.23	EC 2.3.3.1	zma00020, zma00630, zma01100, zma01110
	B6T856	cl2158_1; LOC100282503 (EC:1.1.1.27); K00016 L-lactate dehydrogenase [EC:1.1.1.27]	1.2	0.22	zma:100282503	zma00010, zma00270, zma00620, zma00640, zma01100, zma01110
	B6TJB6	Proteasome subunit alpha type 5	1.23	0.22	zma:100283546	zma03050
	C0HGV5	Putative uncharacterized protein (Pfam: enolase domain)	1.24	0.31	NF	NF
	B6TJM5	26S protease regulatory subunit 6A	1.26	0.02	NF	NF
	B4FZV6	Putative uncharacterized protein	1.26	0	NF	NF
	C0HF77	Uncharacterized LOC100304374	1.29	0.09	zma:100304374	NF
	B6TRW8	Dihydrolipoyllysine-residue succinyltransferase component of 2-oxoglutarate dehydrogenase complex	1.32	0	zma:100284269	zma00020, zma00310, zma01100, zma01110
	B6TMX0	Pyruvate kinase, cytosolic isozyme (EC:2.7.1.40)	1.32	0.07	zma:100283899	zma00010, zma00230, zma00620, zma00710, zma01100, zma01110
	B8A2W6	pco071606; LOC100280280; K01412 mitochondrial processing peptidase [EC:3.4.24.64]	1.34	0.31	zma:100280280	NF
	C0PGM6	Uncharacterized LOC100383510; K03064 26S proteasome regulatory subunit T4	1.37	0.04	zma:100383510	zma03050
	B6UAK0	umc2374; LOC100285843 (EC:3.1.1.31); K01057 6-phosphogluconolactonase [EC:3.1.1.31]	1.49	0.3	zma:100285843	zma00030, zma01100, zma01110
	B8A0Q6	TIDP3457; LOC100279930; K00234 succinate dehydrogenase (ubiquinone) flavoprotein subunit [EC:1.3.5.1]	1.51	0.04	zma:100279930	zma00020, zma00190, zma01100, zma01110
	B6TYM9	cl13598_-2; LOC100284911; K01365 cathepsin L [EC:3.4.22.15]	1.61	0.04	zma:100284911	zma04145
	B6TLR1	Vignain	2.25	0.06	NF	NF
Cellular homeostasis (GO:0019725)	Q5EUE1	PDIL1-1; protein disulfide isomerase; K09580 protein disulfide-isomerase A1 [EC:5.3.4.1]	0.86	0.14	zma:606409	zma04141
	Q5EUD6	pdi7, PDIL2-2; protein disulfide isomerase7; K09584 protein disulfide-isomerase A6 [EC:5.3.4.1]	1.17	0.22	zma:606414	zma04141
	C4J5G3	Uncharacterized LOC100501719; K00382 dihydrolipoamide dehydrogenase [EC:1.8.1.4]	1.19	0.21	zma:100501719	zma00010, zma00020, zma00260, zma00280, zma00620, zma01100, zma01110
	Q5EUD7	pdi6, PDIL2-1; protein disulfide isomerase6; K01829 protein disulfide-isomerase [EC:5.3.4.1]	1.32	0.25	zma:606413	zma04141
	B4FFQ0	Uncharacterized LOC100193620 T(Pfam: thioredoxin motif)	2.8	0.18	zma:100193620	NF
Carbohydrate metabolic process (GO:0005975)	Q9SPJ9, Q9SPK0	Cell wall invertase 2	0.33	0.05	EC 3.2.1.26	zma00052, zma00500, zma01100
	Q43359	TIDP3317, gpc4; LOC100037774; K00134 glyceraldehyde 3-phosphate dehydrogenase [EC:1.2.1.12]	0.62	0.04	zma:100037774	zma00010, zma01100, zma01110
	Q43706	Sus1 protein	0.82	0.16	EC 2.4.1.13	zma00500, zma01100
	B7ZWY9	Putative uncharacterized protein (Pfam: citrate synthase domain)	1.18	0.23	EC 2.3.3.1	zma00020, zma01100, zma01110, zma00630
	B6T856	cl2158_1; LOC100282503 (EC:1.1.1.27); K00016 L-lactate dehydrogenase [EC:1.1.1.27]	1.2	0.22	zma:100282503	zma00010, zma00270, zma00620, zma00640, zma01100, zma01110
	C0HGV5	Putative uncharacterized protein (Pfam: enolase domain)	1.24	0.31	NF	NF
	Q9LLB8	Exoglucanase	1.27	0.2	EC 3.2.1.91	NF
	B6TMX0	Pyruvate kinase, cytosolic isozyme (EC:2.7.1.40); K00873 pyruvate kinase [EC:2.7.1.40]	1.32	0.07	zma:100283899	zma00010, zma00230, zma00620, zma00710, zma01100, zma01110
	B4FFJ4	Similar to alpha-galactosidase from *Oryza sativa* (Q9FXT4)	1.42	0.09	EC 3.2.1.22	zma00052, zma0056, zma00600, zma00603
	B6UAK0	umc2374; LOC100285843 (EC:3.1.1.31); K01057 6-phosphogluconolactonase [EC:3.1.1.31]	1.49	0.3	zma:100285843	zma00030, zma01100, zma01110
	B4G1X1	Uncharacterized LOC100274554; K07407 alpha-galactosidase [EC:3.2.1.22]	1.59	0.05	zma:100274554	zma00561, zma00600, zma00603

KEGG pathway identifiers: zma00010:glycolysis/gluconeogenesis; zma00020:citrate cycle (TCA cycle); zma00030:pentose phosphate pathway; zma00052:galactose metabolism; zma00190:oxidative phosphorylation; zma00230:purine metabolism; zma00260:glycine, serine, and threonine metabolism; zma00270:cysteine and methionine metabolism; zma00280:valine, leucine, and isoleucine degradation; zma00310:lysine degradation; zma00380:tryptophan metabolism; zma00480:glutathione metabolism; zma00500:starch and sucrose metabolism; zma00561:glycerolipid metabolism; zma00600:sphingolipid metabolism; zma00603:glycosphingolipid biosynthesis; zma00620:pyruvate metabolism; zma00630:glyoxylate and dicarboxylate metabolism; zma00640:propanoate metabolism; zma00710:carbon fixation in photosynthetic organisms; zma01100:metabolic pathways; zma01110:biosynthesis of secondary metabolites; zma03050:proteasome; zma04141:protein processing in endoplasmic reticulum; zma04145:phagosome; zma04146:peroxisome.

The KEGG mapper (http://www.genome.jp/kedd/mapper.html) was used to investigate the metabolic pathways of proteins grouped in catabolic process, cellular homeostasis and carbohydrate metabolic process. As shown in Table 3, the catabolic process involves mainly proteins that are related to the biosynthesis of secondary metabolites, cellular homeostasis including enzymes related to protein processing in ER and carbohydrate metabolic process; and glycolysis and gluconeogenesis processes. For the catabolic process, increased levels of proteins were related to proteasome, as well as proteases such as 26S protease regulatory subunit 6A, mitochondrial processing peptidase, and K01365 cathepsin L. Proteome and transcriptome studies in developing seeds of *Medicago truncatula* show transcriptional up-regulation for proteins that may play a role in protein degradation either during reserve accumulation or maturation phase (Gallardo et al., 2007). Similar proteome analyses by (Méchin et al., 2007) reported an important function of turnover of proteins that was associated with the switch from growth and differentiation to storage in maize. In *mn*1 seeds, the presence of proteases in an early stage of development suggests important protein turnover and processing taking place in the BETL region. Interestingly, a leucine aminopeptidase, known to participate in the glutathione metabolism, was increased in the mutant. These data indicate a role of potential redox regulation mediated by glutathione in the BETL enriched basal endosperm.

The cellular homeostasis group was mainly composed of protein disulfide isomerases (PDIs), which showed increased ratios in the *mn*1 when compared with the *Mn*1. Genome-wide search has shown 22 PDI-like sequences in maize (Houston et al., 2005), and the PDIs are known to act as molecular chaperons that contain thioredoxin domains critical in the formation of proper disulfide bonds during protein folding. Thus, the increased abundance of PDI protein in the *mn*1 mutant was consistent with its proposed role in ER stress resulting from reduced Golgi in the BETL of the *mn*1 than the *Mn*1 (Kang et al., 2009). Interestingly, an uncharacterized protein with a thioredoxin motif (B4FFQ0) (Table 3) showed a 2.8-fold increase in *mn*1, suggesting an up-regulation of the gene essential in the folding of new proteins as well as in response to the oxidation/reduction generated by metabolic stress in the mutant lacking INCW2 (Freedman et al., 1994; Houston et al., 2005). These two groups of proteins may have complementary functions in response to the lack of glucose/fructose in the *mn*1 basal endosperm, described previously (LeClere et al., 2010; Chourey et al., 2012).

Carbohydrate metabolism and glycolysis process are highly regulated in seeds. For examples, Gallardo et al. (2007) reported in *M. truncatula* seeds, the genes involved in glycolysis are differentially expressed in seed development, meanwhile those involved in starch synthesis are transiently expressed in seed filling. In the case of the carbohydrate metabolic process group, cell wall invertase 2 was reduced in the *mn*1, as expected. In addition, Sus1 protein was also decreased in the *mn*1. Although the conversion of sucrose to hexose via SUS uses half of the ATP in comparison to the invertase route, the SUS reaction is reversible and is sensitive to the hexose phosphate levels (Barratt et al., 2009), suggesting a possible connection between SUS and invertase in maize BETL region. Physiological roles of these two sucrolytical enzymes are well studied in the transfer cells of developing seeds in cotton (Ruan Y-L Llewellyn and Furbank, 2003; Pugh et al., 2010) and maize (Kang et al., 2009). The cytosolic glyceroldehyde-3-phosphate dehydrogenase GAPDH in the *mn*1 was reduced by one half, suggesting low flux into the glycolysis pathway due to the lack of glucose/fructose in the BETL region (LeClere et al., 2010; Chourey et al., 2012). Another level of regulation can be related to the thioredoxin-like protein. Reoxidation of glyceroldehyde-3-phosphate dehydrogenase through oxidized thioredoxin seems to be a conserved mechanism for protecting the cells from oxidative stress. GAPDH catalyzes the reversible oxidative phosphorylation of glyceraldehide-3-phospate to 1, 3-biphosphoglycerate. A reduction in its activity results in the accumulation of dihydroxyacetone phosphate, which triggers the repression of glycolysis-related enzymes and certain enzymes of the Pentose Phosphate pathway (PPP) are induced as well. PPP enzymes are critical in the maintenance of cytoplasmic NADPH necessary for the antioxidant systems such as glutathione and thioredoxin (Ralser et al., 2007; Neilson et al., 2011). In plants, the GAPDH enzyme is inactivated to stop consumption of ATP for starch synthesis, and starch hydrolysis takes place for producing fuel for the plants (Nelson and Cox, 2008). A 6-phosphogluconolactonase like protein [EC: 3.1.1.31] (B6UAK0) (Table 3), known to participate in the PPP according to the KEGG, result was increased, supporting the hypothesis of protection from oxidative stress in the *mn*1 kernel.

### Secretory proteins in the BETL

As indicated previously, one of the main functions of the basal part of maize endosperm is acquisition and transfer of nutrition and water from maternal plant to developing seed. As in all transfer cells, the main feature of the BETL is the labyrinth-like WIG that increases the plasma membrane area comprised of cellulosic molecules, and enhances transport capacity in these cells (Pugh et al., 2010). In maize, these cells are particularly rich in endoplasmic reticulum (ER), Golgi, mitochondria, and secretory functions. In fact, many of these functions are greatly altered in the *mn*1 mutant due to the loss of the INCW2 (Kang et al., 2009). Therefore, we focused here in the identification of some of the proteins that showed the highest probability of localization in the secretory pathway (Table 4). Not surprisingly, the *Mn*1-encoded INCW2 was found with the highest score as it has an N-terminal secretory signal peptide (Taliercio et al., 1999). The results of the immunogold labeling of the ER and the secretory organelles are consistent with the notion that INCW2 is synthesized by ER-bound ribosomes and delivered to the WIG via the Golgi and TGN compartments (Kang et al., 2009).

**Table 4:** **Proteins predicted to have a signal peptide for the secretory pathway using TargetP**.

**Accession**	**Name**	**SPs**		**CWAPs**		**Len**	**SP**	**Loc**	**RC**
		***mn*1/*Mn*1**	***SD***	***mn*1/*Mn*1**	***SD***				
Q9SPK0	Cell wall invertase 2	0.33	0.05	0.33	0.08	592	0.99	S	1
C0HF77	Putative uncharacterized protein	1.29	0.09	1.78	0.12	778	0.95	S	1
B6U0V6	Endoplasmin	1.18	0.21	1.44	0.11	807	0.90	S	1
B6SHX0	5a2 protein	0.46	0.00	0.53	0.02	107	0.86	S	2
C5JA67	BETL-9 protein	0.45	0.01	0.49	0.00	107	0.74	S	2
B4FG65	Similar to catalytic/hydrolase from *Zea mays* (B6U0A3)	2.63	0.28	2.42	0.04	360	0.69	S	3
Q5EUD7	Protein disulfide isomerase	1.32	0.25	ND	ND	367	1.00	S	1
Q5EUE1	Protein disulfide isomerase	0.86	0.14	ND	ND	514	0.99	S	1
B6TNF1	Calnexin	1.11	0.18	ND	ND	534	0.99	S	1
Q5EUD6	Protein disulfide isomerase	1.17	0.22	ND	ND	366	0.99	S	2
B6TYM9	Vignain	1.61	0.04	ND	ND	376	0.99	S	1
B6UAU9	Rhicadhesin receptor	1.35	0.17	ND	ND	218	0.95	S	1
B4FFJ4	Similar to alpha-galactosidase from *Oryza sativa* (Q9FXT4)	1.42	0.09	ND	ND	423	0.95	S	1
B4G218	Putative uncharacterized protein	1.87	0.52	ND	ND	353	0.94	S	1
B4G1X1	Putative uncharacterized protein	1.59	0.05	ND	ND	423	0.94	S	1
B6TVW2	Germin-like protein subfamily 1 member 17	1.49	0.18	ND	ND	226	0.94	S	1
Q9LLB8	Exoglucanase	1.27	0.20	ND	ND	622	0.81	S	2
B6TLR1	Vignain	2.25	0.06	ND	ND	377	0.78	S	2
Q43712	Calcium-binding protein	ND	ND	1.26	0.09	421	1.00	S	1
B6TDW7	Secretory protein	ND	ND	0.63	0.04	228	0.99	S	1
C0PDM0	Similar to vaculor H+-pyrophosphatase from *Sorghum bicolor* (D9IG65)	ND	ND	0.72	0.03	762	0.99	S	1
B4FSK9	Peroxidase 1	ND	ND	1.85	0.53	362	0.97	S	1
B4FSU9	Hydrolase, hydrolyzing O-glycosyl compounds	ND	ND	0.68	0.01	576	0.96	S	1
Q4FZ46	Cystatin	ND	ND	0.51	0.05	127	0.95	S	1
Q4FZ49	Putative cystatin	ND	ND	0.72	0.05	129	0.95	S	3
B4FX40	Similar to cysteine proteinase 1 from *Zea mays* (Q10716)	ND	ND	1.59	0.04	371	0.94	S	1
B8A0J2	Putative uncharacterized protein	ND	ND	1.17	0.06	897	0.93	S	1
B6T6D4	Peroxidase 27	ND	ND	0.53	0.01	355	0.91	S	3
Q9M588	Prohibitin	ND	ND	1.25	0.07	284	0.89	S	1
Q946V2	Legumin 1	ND	ND	0.60	0.01	483	0.81	S	2
Q5EUD5	Protein disulfide isomerase	ND	ND	1.31	0.12	439	0.80	S	2
Q948J8	Uncleaved legumin-1	ND	ND	0.63	0.02	482	0.77	S	3
B6THU9	Peroxidase 39	ND	ND	0.78	0.03	328	0.77	S	4
B4G004	Similar to beta-glucosidase 7 from *Oryza sativa* (Q75I93)	ND	ND	0.62	0.02	502	0.76	S	3
B6TPA2	Putative uncharacterized protein	ND	ND	0.63	0.08	390	0.67	S	3
B6TC70	Acid phosphatase	ND	ND	1.69	0.24	524	0.57	S	4
B4F7T9	Putative uncharacterized protein	ND	ND	0.75	0.01	354	0.28	S	5

Of the 37 secretory proteins listed in the Table 4, only the INCW2 and the BETL9 are previously reported to be associated with the BETL. Although the BETL9 is expressed abundantly at the RNA level (Gómez et al., 2009 and Table 5) and was greatly reduced in the *mn*1 at both RNA (Xiong et al., 2011) and protein levels, nothing is known about its function in seed development. Calnexin (B6TNF1), endoplasmin (B6U0V6), calreticulin (Q43712), PDIL1-1 (Q5EUE1), PDI7 (Q5EUD6), PDI8 (Q5EUD5), and a putative uncharacterized protein with a domain of HSP70 (B8A0J2) are actively involved in the protein processing of ER pathway. There were some proteins that participate in the protein recognition by the luminal chaperones, lectin related proteins and terminal misfolded targeting protein to the proteasome (Table S7). Prohibitin (Q9M588) was identified with high probability of localizing to the secretory pathway, with ratios of 1.28 and 1.32 in the two experiments. This protein is predicted to function in mitochondrial processes including protein processing, respiratory chain function and mitochondrial DNA organization (Van Aken et al., 2010). Cystatins (Q4FZ46) are proteins that inhibit cysteine proteases by binding of cystatins, and are believed to be involved in multiple functions, including biotic and abiotic stress tolerance, programed cell death, and the regulation of various metabolic processes through processing and degradation of various proteins (Massonneau et al., 2005). Recently, van der Linde et al. (2012) demonstrated that cystatin suppresses host immunity by inhibiting apoplastic cysteine proteases that play a pivotal role in defense signaling in maize. Clearly, detailed functional characterization of these novel secretory proteins in basal endosperms is an exciting future direction of research.

**Table 5:** **A comparative profile of RNA and protein levels of select proteins in the *Mn*1 and *mn*1 basal endosperms**.

**Gene number**	**Accession number**	**Genes**		***AV***	***SD***	**ρ**	**qPCR ratio *mn*1/*Mn*1**	**iTRAQ ratio *mn*1/*Mn*1**
1	Q9SPK0	*Mn* 1	*Mn*1	4305	235	<0.01	0.01	0.39
			*mn*1	29	4			
2	Q41779	*BETL* 1	*Mn*1	35752	4649	<0.01	0.12	ND
			*mn*1	4229	6			
3	Q4FZ46	*Psei* 8	*Mn*1	999	62	<0.01	0.15	0.48
			*mn*1	151	8			
4	C5JA67	*BETL* 9	*Mn*1	34925	7122	<0.01	0.21	0.49
			*mn*1	7471	22			
5	B6SHX0	*5a* 2	*Mn*1	17863	5116	<0.01	0.11	0.55
			*mn*1	2047	152			
6	B6TDW7	*Secretory protein*	*Mn*1	537	34	<0.01	0.13	0.58
			*mn*1	69	1			
7	Q946V2	*Legumin*	*Mn*1	24393	2409	<0.01	0.56	0.59
			*mn*1	13729	925			
8	Q5EUE1	*PDI* 1	*Mn*1	10797	841	<0.01	0.53	0.75
			*mn*1	5689	1102			
9	C0PLF0	*PDI* 2	*Mn*1	891	27	<0.01	0.43	0.83
			*mn*1	379	52			
10	C0P5R8	*Amidase*	*Mn*1	508	59	<0.01	0.28	0.85
			*mn*1	140	8			
11	B6TT94	*Hypothetical protein*	*Mn*1	2016	199	<0.01	0.89	0.85
			*mn*1	1795	148			
12	B4F861	*IAA-amino acid hydrolase*	*Mn*1	1021	131	<0.01	0.93	0.88
			*mn*1	948	128			
13	C0HFV7	*Apyrase*	*Mn*1	1972	148	<0.01	0.29	1.18
			*mn*1	572	46			
14	B8A0J2	*Hypothetical protein*	*Mn*1	1668	87	<0.01	0.66	1.23
			*mn*1	1097	99			
15	Q9M588	*Prohibitin* 2	*Mn*1	1131	12	<0.01	0.20	1.28
			*mn*1	230	4			
16	B6U0V6	*Endoplasmin*	*Mn*1	729	126	≤q 0.05	0.70	1.51
			*mn*1	507	5			
17	Q43712	*Calcium-binding protein*	*Mn*1	4707	619	<0.01	0.46	1.20
			*mn*1	2160	81			
18	Q5EUD5	*PDI* 8	*Mn*1	2084	290	<0.01	0.60	1.36
			*mn*1	1258	25			
19	B4FX40	*Cysteine proteinase* 1	*Mn*1	8206	369	<0.01	0.73	1.61
			*mn*1	6027	244			
20	C0HF77	*Hypothetical protein*	*Mn*1	120	20	≥q 0.01	2.46	1.72
			*mn*1	295	44			

Data are presented as the mean of ±SD of six q-PCR analyses (three technical replicates of two biological samples).

AV, represents average # transcripts/ng total RNA.

### RNA and protein abundance of select proteins in the basal endosperm

Our previous studies have established that the *Mn*1 encoded INCW2 as specific biomarker in the BETL, and its expression is greatly reduced in the *mn*1 mutant (Cheng et al., 1996; Chourey et al., 2006). Our proteomic data here (Tables 1, 2) are consistent with the previous results. Transcriptome analyses of the BETL led to a large catalog of genes that showed transcript abundance in the *Mn*1 basal endosperm (Xiong et al., 2011). Table 5 shows results from a comparative analysis of expression of a few select genes at RNA level by q-PCR and by iTRAQ (Table 1) in these two genotypes. There was a qualitative concordance between RNA and the cognate protein levels for the first 10 of the 20 genes that showed *mn*1 to *Mn*1 ratios of <1.0. The ratios between *mn*1:*Mn*1 at both levels of expression were similar, <1.0, but the quantitative values were divergent. In contrast, the expression levels of the other seven genes (13–19) were highly divergent. The qPCR values were <1.0, but the iTRAQ ratios were >1.0. Only one gene (#20) showed a higher ratio in qPCR results than in the iTRAQ results. Two genes, #11 and #12, showed similar ratios of transcript and protein levels in the two genotypes. Overall, our data showed a high level of non-concordance between RNA and protein level expression. Such discrepancy is also described previously; Hajduch et al. (2010) showed an overall concordance of only 56% between RNA and protein levels in developmental seeds of Arabidopsis. It is clear that changes in RNA abundance may not reflect protein level changes due to posttranscriptional and posttranslational regulatory processes in the cells (Gallardo et al., 2007; Lan et al., 2011).

## Conclusions

Transfer cells are ubiquitous in plants, fungi, and animal cells, and are well recognized for their role in solute acquisition and transport functions. Emerging evidence suggests that BETL plays a critical role in seed development (Kang et al., 2009; Pugh et al., 2010; Costa et al., 2012). Transcriptomic analyses in maize BETL identified important genes related to mitochondrial and stress related functions (Xiong et al., 2011). As shown in this study, transcriptomic changes do not directly translate into the changes and functions of proteins, which determine phenotypes. Here we provide information at the proteome level on the proteins and processes in BETL enriched tissues that are targets of the *Mn*1 mutation.

Using iTRAQ LC-MS/MS, we have identified over 2500 proteins and more than 130 differential proteins in the BETL enriched basal endosperm of *mn*1. A large array of diverse functions, including energy and carbohydrate metabolism, secondary metabolism and defense related processes were revealed. Highly regulated protein folding/degradation and redox control may function to maintain the cellular homeostasis. Furthermore, the levels of many secretory proteins were greatly altered in the *mn*1. These results have not only improved our understanding of molecular mechanisms underlying the *mn*1 phenotype, but also laid a foundation for further studies on characterizing novel proteins. Future studies on many of these proteins will greatly enhance our knowledge on resource allocation and tolerance to biotic and abiotic stress in developing seeds.

### Conflict of interest statement

The authors declare that the research was conducted in the absence of any commercial or financial relationships that could be construed as a potential conflict of interest.

## Supplementary materials

Figure S1 **Interactive graph for the analysis of enriched biological functions in (A) SPs and (B) CWAPs with REVIGO tool kit**.

Table S1 **Gene accession numbers and the primers for genes investigated in the present study**.

Table S2 **Total protein ID of soluble proteins**.

Table S3 **Total protein ID of cell wall associated proteins**.

Table S4 **agriGO results for soluble proteins**.

Table S5 **agriGO results for cell wall associated proteins**.

Table S6 **Shared differentially expressed proteins in SPs and CWAPs**.

Table S7 **KEGG identifiers for secretory proteins from TargetP**.
