# Supplementary figures and images for: Proteomic comparison of basal endosperm in maize miniature1 mutant and its wild-type Mn1
Source: Front Plant Sci. 2013 Jun 25;4:211. doi: 10.3389/fpls.2013.00211 (PMC3691554; doi:10.3389/fpls.2013.00211)

**A**

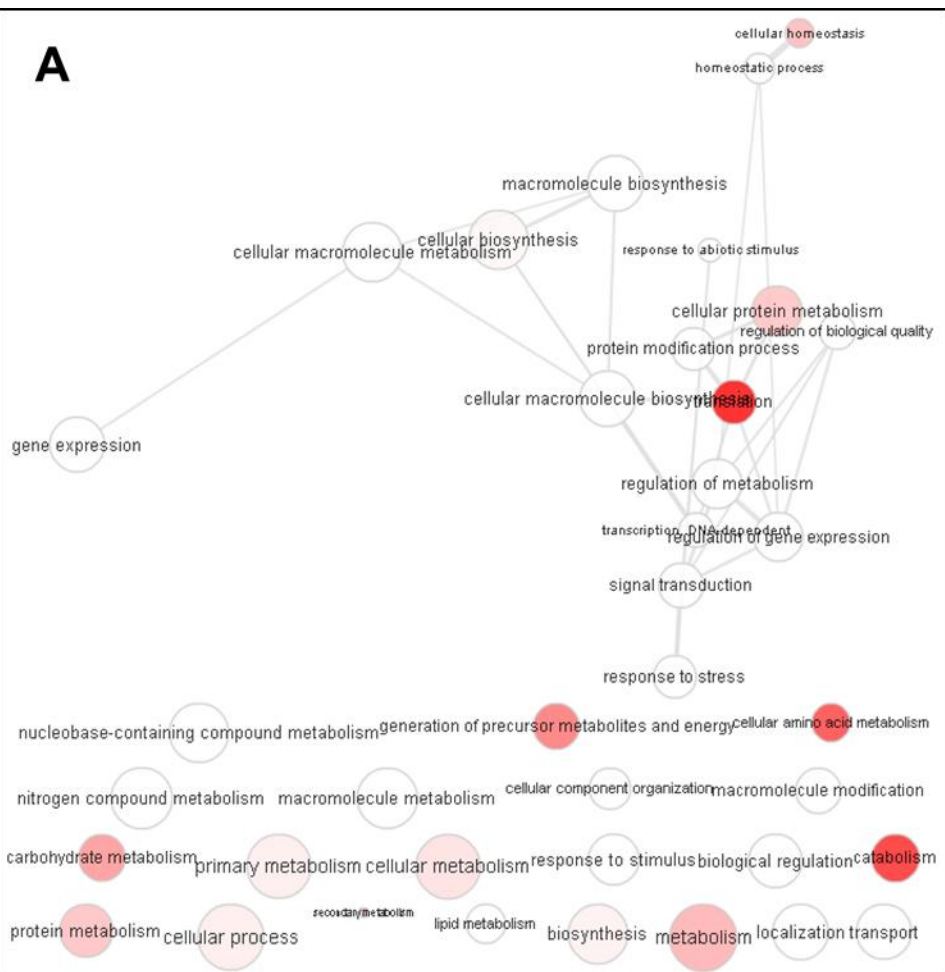

**B**

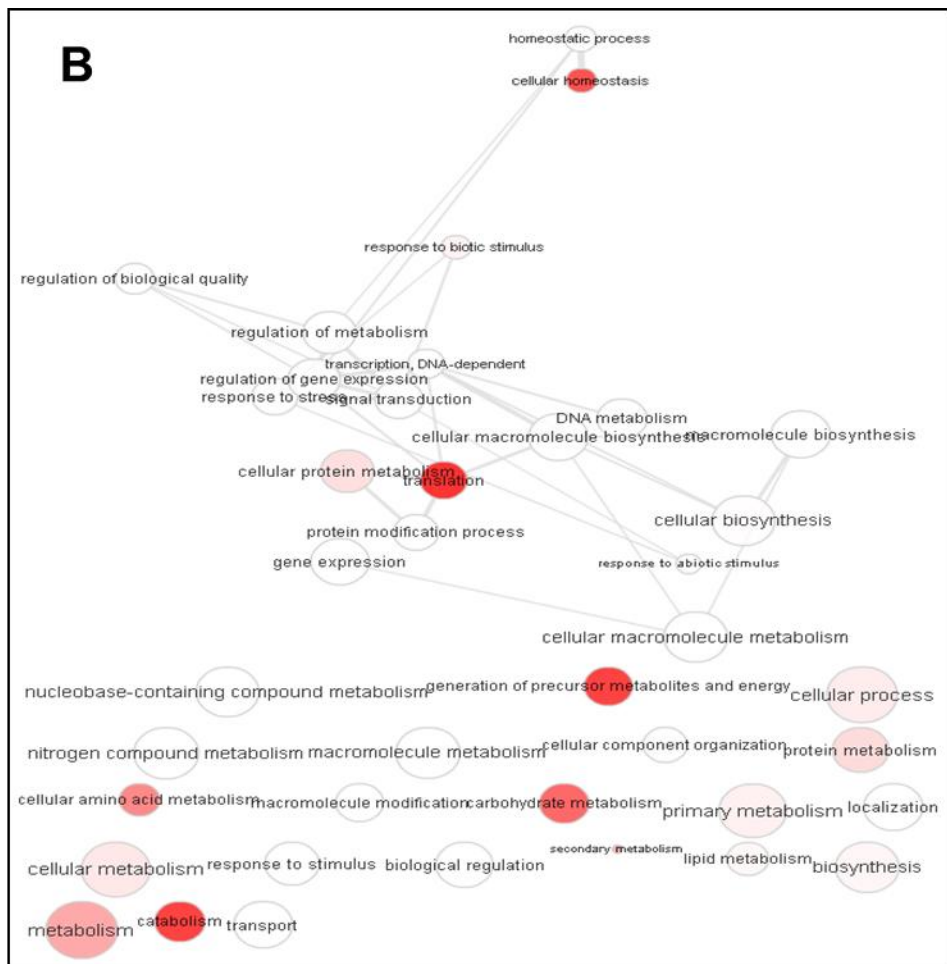

Supplement: Figure S1 — Interactive graph for the analysis of enriched biological functions in (A) SPs and (B) CWAPs with REVIGO tool kit. [file Presentation1.PDF]
